# Improved Chemotherapeutic Activity by *Morus alba* Fruits through Immune Response of Toll-Like Receptor 4

**DOI:** 10.3390/ijms161024139

**Published:** 2015-10-13

**Authors:** Bo Yoon Chang, Seon Beom Kim, Mi Kyeong Lee, Hyun Park, Sung Yeon Kim

**Affiliations:** 1 Institute of Pharmaceutical Research and Development, College of Pharmacy, Wonkwang University, Iksan, Jeonbuk 570-749, Korea; E-Mail: oama611@nate.com; 2 College of Pharmacy, Chungbuk National University, Cheongju 361-763, Korea; E-Mails: suntiger85@hanmail.net (S.B.K.); mklee@chungbuk.ac.kr (M.K.L.); 3 Institute of Zoonosis Research Center and Department Infection Biology, College of Medicine, Wonkwang University, Iksan, Jeonbuk 570-749, Korea; E-Mail: hyunpk@wonkwang.ac.kr

**Keywords:** *Morus alba* fruits, immune response, TLR, 5-fluorouracil (5-FU), chemotherapeutic activity

## Abstract

*Morus alba* L. fruits have long been used in traditional medicine by many cultures. Their medicinal attributes include cardiovascular, hepatoprotective, neuroprotective and immunomodulatory actions. However, their mechanism of macrophage activation and anti-cancer effects remain unclear. The present study investigated the molecular mechanisms of immune stimulation and improved chemotherapeutic effect of *M. alba* L. fruit extract (MFE). MFE stimulated the production of cytokines, nitric oxide (NO) and tumor necrosis factor-α (TNF-α) and tumoricidal properties of macrophages. MFE activated macrophages through the mitogen-activated protein kinase (MAPKinase) and nuclear factor-κB (NF-κB) signaling pathways downstream from toll-like receptor (TLR) 4. MFE was shown to exhibit cytotoxicity of CT26 cells via the activated macrophages, even though MFE did not directly affect CT26 cells. In a xenograft mouse model, MFE significantly enhanced anti-cancer activity combined with 5-fluorouracil and markedly promoted splenocyte proliferation, natural killer (NK) cell activity, cytotoxic T lymphocyte (CTL) activity and IFN-γ production. Immunoglobulin G (IgG) antibody levels were significantly increased. These results indicate the indirect anti-cancer activity of MFE through improved immune response mediated by TLR4 signaling. *M. alba* L. fruit extract might be a potential anti-tumor immunomodulatory candidate chemotherapy agent.

## 1. Introduction

Cancer treatment strategies include surgery, chemotherapy, and radiation therapy. Many cancer chemotherapeutic agents can produce toxicity, which particularly leads to myelosuppression and immunosuppression [1]. Immune status is important for growth and the combatting of cancer.

Enhancement of host immune response has been accepted as a tool for defense against cancer without any side effects. Effective immune enhancement for anti-tumor activity requires antigen presenting cells, T lymphocytes, and natural killer (NK) cells [2,3]. Many candidate cancer chemotherapeutic agents activate significant antigen presenting cells, such as dendritic cells and macrophages, through toll-like receptors (TLRs), which are able to perform antigen uptake, processing and initiation of T cell responses. TLRs are very important in early innate immune defense. The TLR agonists have a potential of tumoricidal activity via TLR-activated innate immune responses. Some TLR agonists are being clinically investigated [4,5].

Natural herbs have been used as complementary and alternative medicine for a long time. Among other things, they were used to reduce side effects and enhance tumoricidal effects of chemotherapy in cancer patients. [4,6]. *Morus alba* L. is widely distributed in Asia and has been traditionally used in Korea. Pharmacological functions attributed to *M. alba* L*.* include anti-oxidative, anti-inflammatory, anti-diabetic, and immune stimulating effects, and cardiovascular, hepatoprotective, and neuroprotective actions [7,8]. *M. alba* L*.* fruit contains anthocyanins [9,10], alkaloids [10], flavonoids [11,12], and polysaccharides [13].

Our previous study has shown that *M. alba* L. has immune-stimulatory effects in macrophages [14]. Also, pyrrole alkaloids in *M. alba* fruits having immune-stimulating were reported [15]. However, the underlying mechanism of stimulatory activity remains unclear. To investigate the immune modulatory behavior of *M. alba* fruit, the underlying mechanism of the immune-stimulatory effect was investigated. Also, the enhancement of therapeutic potential with 5-fluorouracil (5-FU) was estimated in a mouse tumor xenograft model.

## 2. Results

### 2.1. Mechanisms of Macrophage Stimulation by M. alba L. Fruit Extract (MFE)

Pattern recognition receptors, such as TLR2, TLR4, and TLR6, are involved in the binding of natural products to macrophages, which leads to macrophage activation [16,17]. To study the effect of Pattern recognition receptor (PRR) blockade, macrophages were incubated with 10, 30, 100 μg/mL of MFE with or without TLR 2, 4 and 6 antibodies for 24 h. Treatment with MFE significantly increased production of nitric oxide (NO) and TNF-α without antibodies (Table 1). The MFE associated increase of NO and TNF-α was significantly decreased by TLR4 antibodies to 73%–82% and 64%–71% of antigen free control, respectively. NO production stimulated by MFE was inhibited only by TLR4 antibodies. TLR2 and TLR6 antibodies did not inhibit NO and TNF-α production. TLR4 antibodies most potently inhibited secretion of TNF-α with secretion inhibited less by TLR2 and TLR6 antibodies (Table 1).

**Figure 1:**
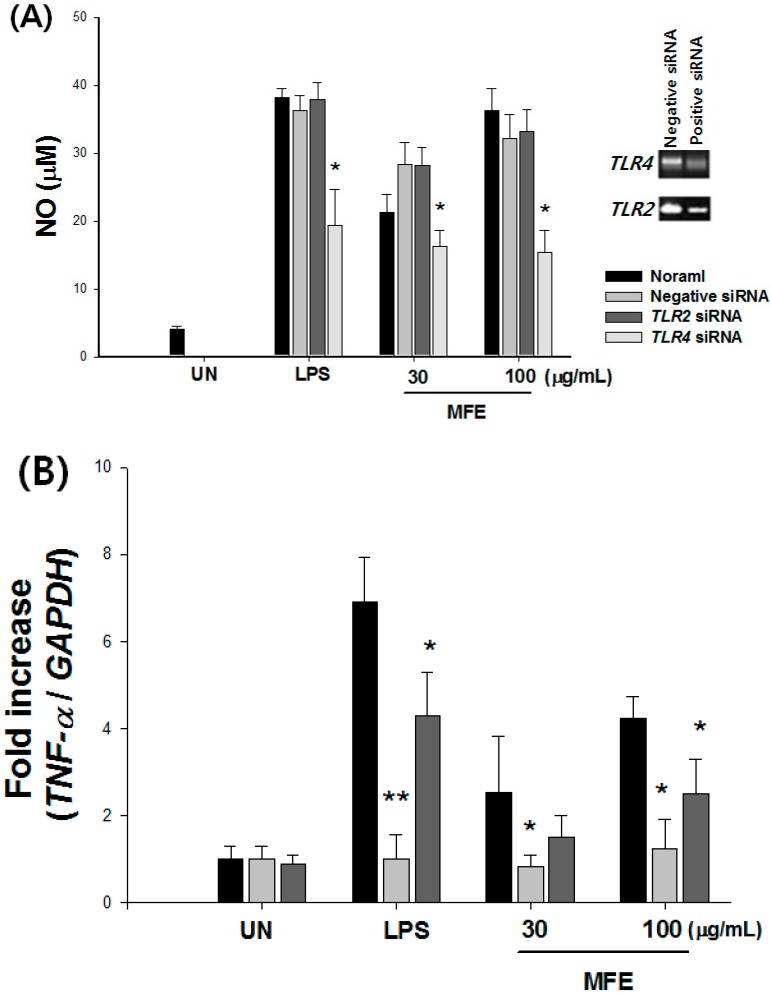

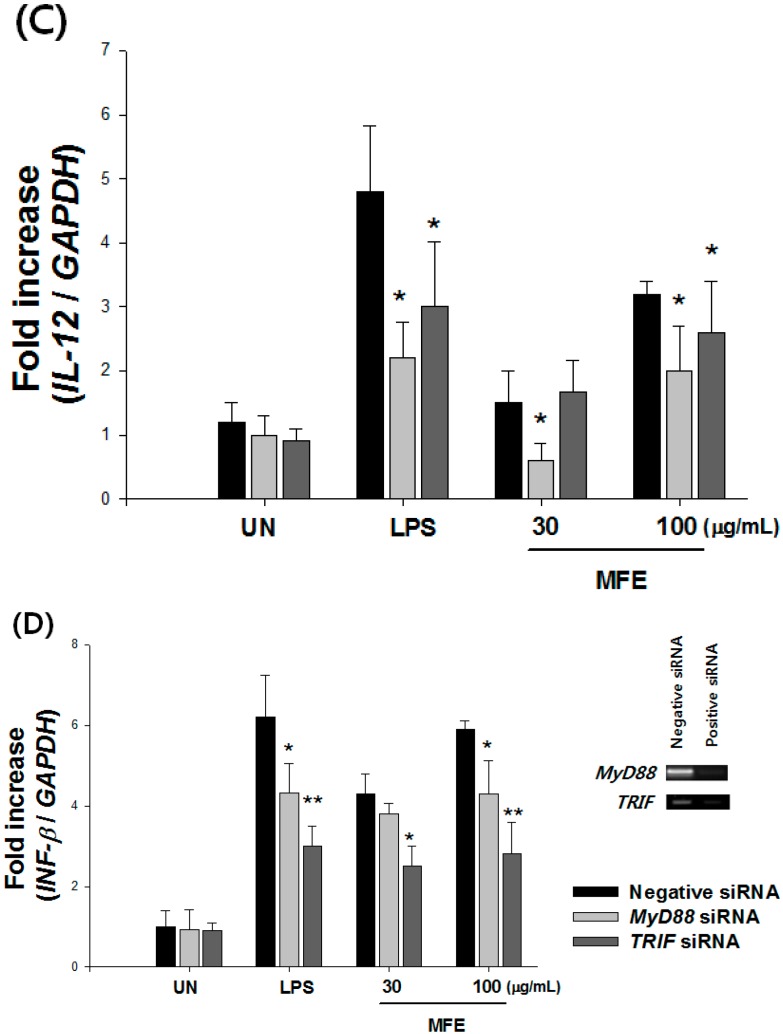
MFE activates macrophages in a TLR4-dependent manner. (**A**) After transfection with TLR2 or TLR4 siRNA, cells were activated with LPS or MFE for 24 h. NO production was determined using Griess reagent. Macrophages were transfected with MyD88 siRNA, TRIF siRNA, or scramble siRNA, respectively. Twenty-four hours after transfection, macrophages were treated with 30 or 100 μg/mL of MFE; (**B**) TNF-α; (**C**) IL-12 and (**D**) IFN-β expression were measured by real-time PCR. The values are presented as means ± S.D. * *p* < 0.05, ** *p* < 0.01, significantly different from the negative siRNA group. MFE: *Morus alba* L. Fruit Extract; TLR: toll-like receptor; LPS: lipopolysaccharide; NO: nitric oxide; TNF-α: tumor necrosis factor-α; TRIF: TIR-domain-containing adapter-inducing interferon-β; IFN-β: interferon-β; UN: untreated.

Gene expressions of *TLR2* and *TLR4* in macrophages were knocked-down by transfection with the respective siRNA. Significantly reduced production of NO was observed in *TLR4* gene knock-down cells (Figure 1A). There was no effect of *TLR2* gene knockdown on NO production. To examine the effects of *MyD88* and TIR-domain-containing adapter-inducing interferon-β (*TRIF*) knockdown on the MFE-triggered TLR4 signaling, the cytokines *TNF-α*, *IL-12* and *IFN-β* in the siRNA-transfected macrophages were measured. *MyD88* and *TRIF* siRNA-transfected macrophages were significantly inhibited by MFE-induced *TNF-α*, *IL-12* and *IFN-β* expression as compared to negative siRNA-transfected macrophages (Figure 1B–D).

**Table 1:** Inhibition of MFE induced NO and TNF-α production by the TLR antibodies in macrophage.

		NO (%)				TNF-α (%)			
		Ab Free	TLR2 Ab	TLR4 Ab	TLR6 Ab	Ab Free	TLR2 Ab	TLR4 Ab	TLR6 Ab
**Untreated**		100.0 ± 2.4	100.0 ± 3.3	100.0 ± 2.6	100.0 ± 2.4	100.0 ± 3.2	100.0 ± 3.6	100.0 ± 2.6	100.0 ± 1.4
**MFE (μg/mL)**	**10**	140.4 ± 22.4	138.3 ± 22.7	115.7 ± 12.6 *	126.2 ± 19.4	131.9 ± 8.0	121.6 ± 5.3	90.6 ± 4.3 *	131.3 ± 7.8
	**30**	312.9 ± 12.3	280.4 ± 13.5	231.6 ± 13.4 *	277.8 ± 14.6	160.4 ± 12.2	150.1 ± 3.4	102.7 ± 4.6 *	150.5 ± 14.5
	**100**	466.4 ± 10.3	392.4 ± 12.5	340.4 ± 8.3 *	340.1 ± 7.3	172.9 ± 3.2	159.6 ± 24.6	122.7 ± 5.3 *	167.2 ± 18.3

MFE: *Morus alba* L. Fruit Extract; NO: nitric oxide; TNF-α: tumor necrosis factor-α; TLR: toll-like receptor. The values were presented as means ± S.D. * *p* < 0.05, significantly different from the Ab-free group.

**Figure 2:**
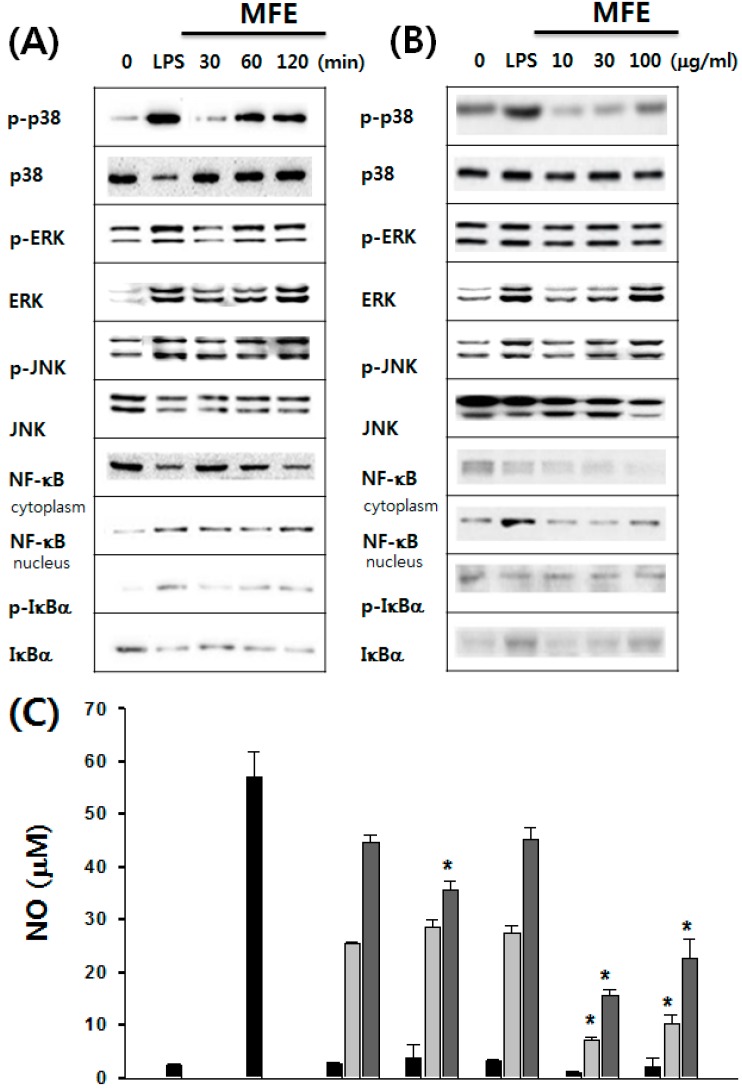

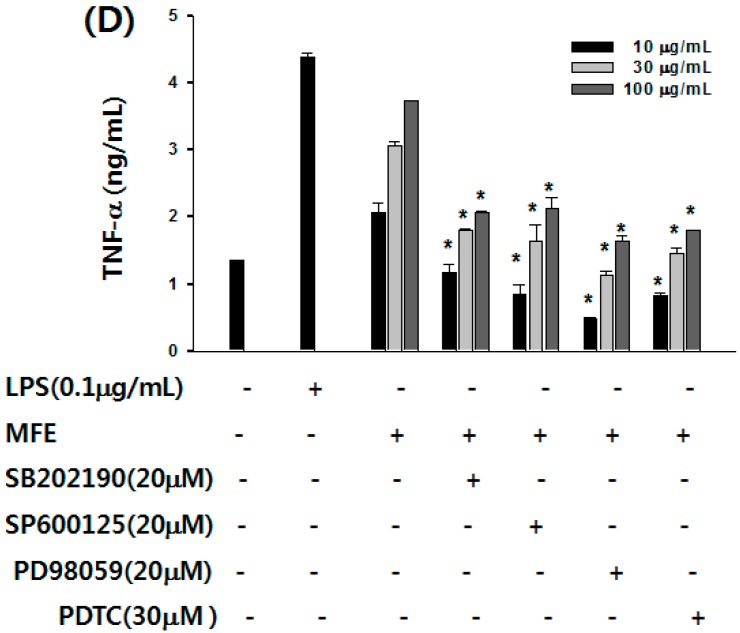
Effect of MFE on MAPK and NF-κB mediating pathways. (**A**) Macrophages were treated with LPS (0.1 μg/mL) or MFE (30 μg/mL) for 0, 30, 60, and 120 min; (**B**) Cells were treated with LPS (0.1 μg/mL) or MFE (10, 30 and 100 μg/mL) for 60 min. Phosphorylated-p38, JNK, ERK and NF-κB were detected by Western blotting with specific antibodies. Macrophages were pretreated with MAPK and NF-κB inhibitors for 2 h in medium. Thereafter, cells treated with MFE were incubated in fresh medium for 24 h. LPS was used as the positive control; (**C**) NO and (**D**) TNF-α production in supernatants were measured using ELISA. The values were presented as means ± S.D. * *p* < 0.01, significantly different from the untreated group. MAPK: mitogen-activated protein kinase; NF-κB: nuclear factor-κ B; JNK: c-jun N-terminal kinase; ERK: extracellular signal-regulated kinase.

We investigated the mechanism of macrophage activation via the mitogen-activated protein kinase (MAPK) by MFE. The phosphorylation of extracellular signal-regulated kinase (ERK), and c-jun N-terminal kinase (JNK) were measured in macrophages. MFE time- and dose-dependently induced phosphorylation of p38, ERK and JNK in macrophages (Figure 2A,B). MFE also increased nuclear factor-κ B (NF-κB) heterodimer (p65/p50 subunits) protein expression in the cytoplasm and nuclear translocation. These results suggest that MFE activates macrophages medicated through MAPK and NF-κB signaling pathways. To confirm the relationship of MAPK and NF-κB activation to MFE, MAPK and NF-κB inhibitors (SB203580 for p38, SP600125 for JNK, PD98059 for ERK, and pyrrolidinedithiocarbamate (PDTC) for NF-κB) in MFE-treated macrophages were investigated. MFE-induced NO production was significantly decreased after treatment with SB203580, PD98059, and PDTC. But, treatment with SP600125 did not affect MFE-induced production of NO (Figure 2C). MFE induced TNF-α production was inhibited significantly after treatment with SB203580, SP600125, PD98059, and PDTC. These results suggest that the p38, ERK, and NF-κB pathways are required for MFE-mediated NO production and that the p38, ERK, JNK MAPK pathways, and NF-κB-mediated pathway are required for MFE-mediated TNF-α production (Figure 2D).

### 2.2. Macrophage Activation of MFE for Tumor Cytotoxicity

**Figure 3:**
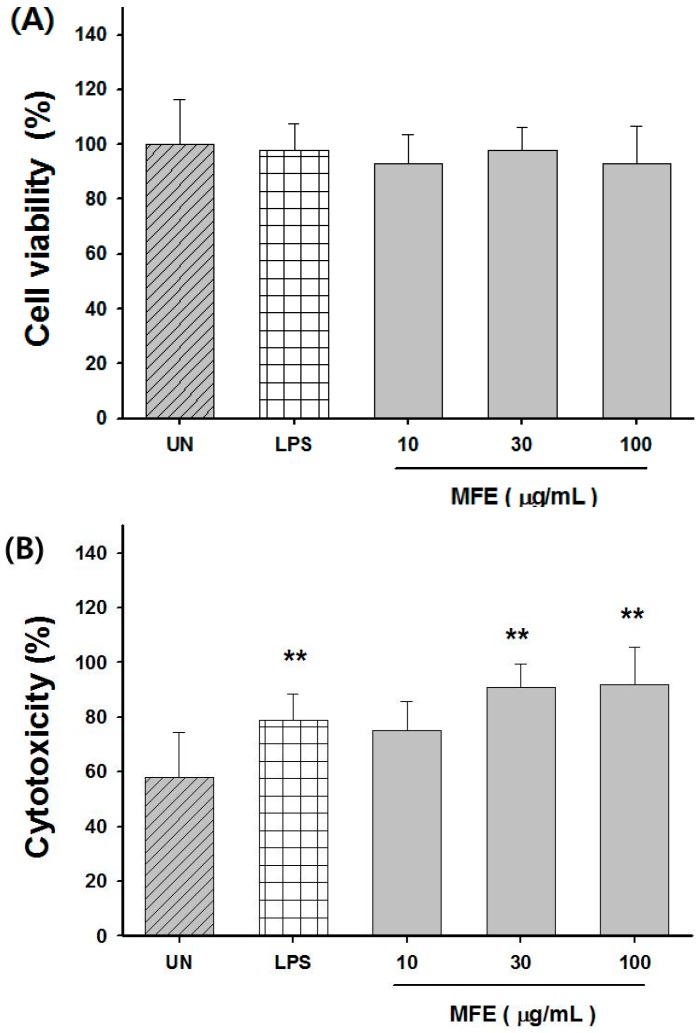
Cytoxicity of MFE *in vitro*. (**A**) The effect of MFE on the proliferation of CT26 tumor cells; (**B**) The effect of MFE on macrophage-mediated cytotoxicity against CT26 tumor cells. Macrophages were activated by MFE and co-cultured with CT26 tumor cells for additional 24 h at the ratio of 10:1 (macrophage to CT26). The values are presented as mean ± S.D. ** *p* < 0.01, significantly different from the untreated group.

Macrophages can attack tumor cells when they are exposed to certain stimuli [18]. We examined the cytotoxicity of MFE in CT26 cells. As shown in Figure 3A, MFE at concentration on 10, 30 and 100 μg/mL did not have any antitumor activity in CT26. However cytotoxicity against CT26 in the group treated with MFE was significantly higher compared to untreated group in peritoneal macrophages (Figure 3B). These results suggested that cytotoxicity against tumor cells by MFE might be associated with activation of macrophages.

### 2.3. Enhancement of Chemotherapeutic Effects by MFE in Xenograft Mice

**Figure 4:**
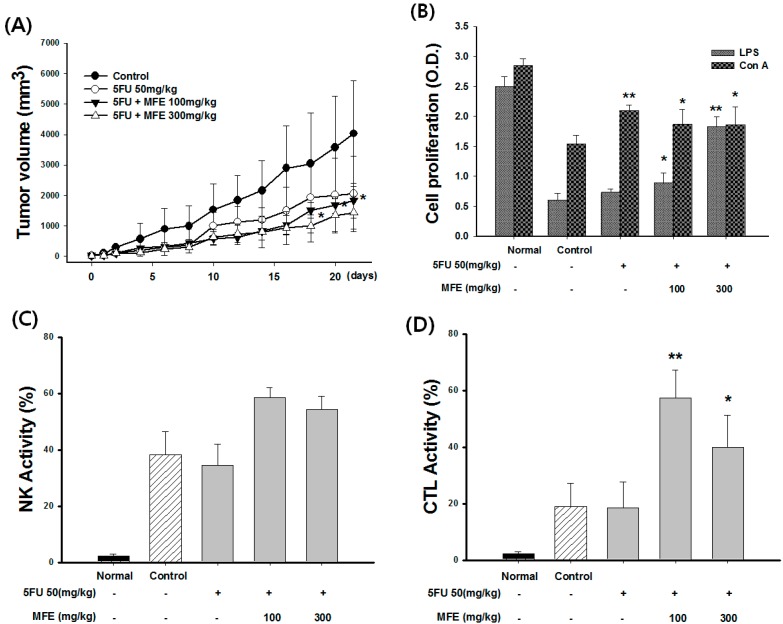

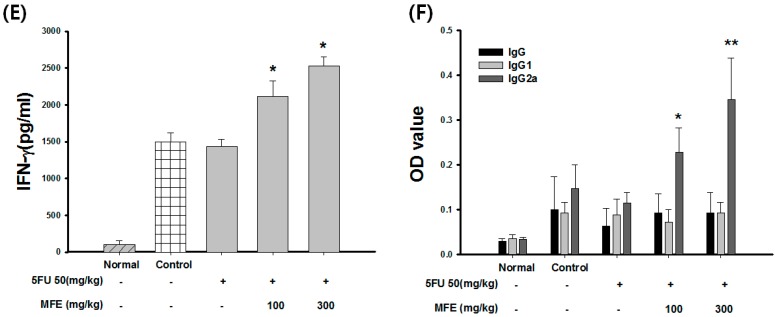
Effect of MFE on cellular and humoral immune response in a mouse xenograft model. (**A**) Changes of tumor volume in the tumor xenograft mice. The values are presented as mean ± S.D. * *p* < 0.05 significantly different from the 5-FU group; (**B**) Splenocyte proliferation. The values are presented as mean ± S.D. * *p* < 0.05, ** *p* < 0.01 significantly different from the control group; (**C**) NK cells and (**D**) CTL activity were measured by the MTT assay. The values are presented as mean ± S.D. * *p* < 0.05, ** *p* < 0.01 significantly different from the control group; (**E**) Contents of IFN-γ in the culture supernatants were determined by ELISA. The values are presented as mean ± S.D. * *p* < 0.05 significantly different from the control group and (**F**) Serum antigen-specific IgG, IgG1, and IgG2a antibodies were measured by ELISA. The values are presented as mean ± S.D. * *p* < 0.05, ** *p* < 0.01 significantly different from the control group. 5-FU: 5-fluorouracil; NK: natural killer; CTL: cytotoxic T lymphocyte.

MFE inhibited the growth of tumor cells in xenograft mice (Figure 4A). MFE 300 mg/kg in combination with 5-fluorouracil (5-FU) significantly reduced the tumor volume more than with 5-FU alone. In this experimental condition, 5-FU showed a 21% inhibitory rate on colon cancer weight. Interestingly, the inhibitory rate was significantly increased in a dose-dependent fashion from 21% to 29% and 42% when 5-FU and MFE were applied together (Table 2).

**Table 2:** Effects of MFE on leukocytes and the growth of tumors in the xenograft mice.

Group	Dose (mg/kg)	Leukocyte (10^3^/mL)	Weight of Spleen (g)	Weight of Tumor (g)	Inhibition Rate of Tumor (%)
Control	-	12.3 ± 1.35	0.45 ± 0.04	5.81 ± 1.35	-
5-FU	50	8.32 ± 1.35	0.32 ± 0.23	4.62 ± 1.24	21
5-FU + MFE	50 + 100	12.5 ± 0.65 *	0.43 ± 0.20	4.26 ± 0.65	29
	50 + 300	14.2 ± 0.95 *	0.52 ± 0.08 *	3.75 ± 0.95	42

The values are presented as mean ± S.D. * *p* < 0.05, significantly different from the 5-FU-only group. 5-FU: 5-fluorouracil.

Leukocyte counts of mice treated with 5-FU group were decreased significantly, while counts increased significantly in mice treated with 5-FU + MFE (Table 2). 5-FU, as an immune suppressor, significantly decreased spleen weight 29% more of than that of the control group. However, high doses of MFE significantly increased the spleen weight as compared to 5-FU groups (*p* < 0.05). MFE alleviated the loss of spleen weight induced by 5-FU (Table 2). Additionally, the stimulating effect on proliferation by MFE was determined using a splenocyte in the tumor xenograft mice (Figure 4B). LPS stimulates B cell proliferation and Concanavalin A (ConA) stimulates T cell proliferation. MFE could significantly enhance ConA- and LPS-induced splenocyte proliferation in the tumor xenograft mice (*p* < 0.05 and *p* < 0.01, respectively). However, the 5-FU group did not display an increase that was significant compared to the control group. These results suggested that MFE might be related to stimulation of T cells and B cells, also humoral and cell-mediated immunity in the tumor xenograft mice. Tumor cell elimination is mediated in part by the cytotoxic activity of NK cells and CTL. However, 5-FU + MFE significantly increased the NK cells and CTL activity in the tumor xenograft mice (*p* < 0.05 and *p* < 0.01, respectively). The effect of MFE on the production of interferon-γ (IFN-γ) in ConA-stimulated splenocytes was measured (Figure 4E). The production of IFN-γ was dramatically increased by MFE (*p* < 0.05). The effect of MFE on humoral immunity was investigated by total IgG and IgG subclasses (IgG1 and IgG2a) in serum. MFE did not affect IgG and IgG1 antibody levels. On the other hand, IgG2a antibody levels were significantly enhanced by treatment with MFE in the tumor xenograft mice (Figure 4F).

## 3. Discussion

TLRs are existent in various cell and tissues. Above all, the role of TLRs is important in macrophage and dendritic cells. TLRs make an important link between the innate and adaptive immune systems because their activation leads to the expression of co-stimulatory molecules of immune cells, which convert them into defective antigen presenting cells. The TLR families are made up of thirteen different TLR molecules which differ in the microbial structures they recognize. TLRs are preferentially expressed on phagocytes, dendritic cells, and epithelial cells at sites at which bacteria enter into the host. The binding of microbial components to the TLRs effectively acts as a signal to increase immune stimulation and allows them to activate T and B lymphocytes [19,20].

The binding of LPS to TLR4 is a critical event in immune activation [21]. MFE and LPS have a similar chemical structure, and they share the same pathways for TLR4 signaling.

*M. alba* fruit is enriched in anthocyanins [9], alkaloids [10], flavonoids [11,12], and some polysaccharides [13,22,23,24]. Potential synergy effects occur in plants or healthy foods due to the complex mixture of various phytochemicals [25]. Therefore, these studies were performed using extracts instead of individual compounds to understand the beneficial effects of *M. alba* fruit extract, specifically the mechanisms of its immune-stimulatory effects and cytotoxicity against cancer through immune responses.

MFE was associated with the release of immune-stimulating cytokines like TNF-α and NO in a dose-dependent manner in macrophages. The blocking of TLR4 antibodies significantly inhibited MFE-induced NO production and TNF-α secretion. Moreover, MFE-induced NO production was attenuated by gene knockdown of TLR4. Therefore, immune activity by MFE may occur through activation of the TLR4 signaling pathway.

The TLR4 signal can use both MyD88 and TRIF pathways in response to gram-negative endotoxins. Why TLR4 signaling uses both of these pathways is unclear [26]. The two different pathways of TLR result in two types of adaptors. The MyD88 pathways induced early phase activation of NF-κB through the interleukin-1 receptor-associated kinase (IRAK), and the IRAK proteins interacted with and activated TNF receptor-associated factor-6 (TRAF6). TRAF6 promotes ubiquitination of downstream signaling mileculesm and activates TGF-β activated kinase 1 (TAK1), which in turn initiates the MAPK and NF-κB. The TRIF pathway requires the TRIF-associated molecule (TRAM) and results in both NF-κB activation and interferon regulatory factor (IRF)-7-dependent synthesis of type I interferon (IFN). MFE-induced TNF-α, IL-12 and IFN-β production were analyzed to investigate the role of both pathways. Knock-down of MyD88 substantially inhibited MFE-induced TNF-α and IL-12 production. Knock-down of TRIF sensitively inhibited MFE-induced IFN-β production. Our results indicate that MFE led to activation of both MyD88-dependent and TRIF-dependent signaling pathways of TLRs. The NF-κB signaling pathway is activated following clustering of the TLRs. NF-κB generally regulates various types of inducible transcriptional promoting in macrophages [27]. NF-κB is presented in the cytoplasm via interaction with the inhibitory protein IκB [28]. Expression of this protein demonstrated that MFE increased the level of NF-κB in macrophages. MFE stimulated NF-κB nuclear activation and IκBα degradation in cytoplasm. Therefore, MFE induced macrophage activation through the NF-κB signaling pathway.

The NF-κB and MAPK pathways were mostly important in macrophage activation by MFE. MAPK pathways are activated by cytokines and a variety of cellular activities [29,30]. MFE increased the phosphorylation of ERK, JNK, and p38 in a time- and dose-dependent manner. MAPK inhibitors and PDTC pretreatment inhibited MAPK and NF-κB signaling, which led to the inhibition of the production of NO and TNF-α. These results suggest that MFE-mediated macrophage activation largely depends on MAPK and NF-κB signaling. Taken together, the results demonstrate that MFE can be a macrophage activator and immune-stimulator through the TLR4 signaling pathway.

There were observed enhanced anti-tumor effects in the tumor xenograft mice. The cytotoxicity of MFE against tumor cells was not direct, but indirect through the activation of macrophages. These results show that the enhanced tumoricidal effect of MFE was mediated macrophage activation.

5-FU is considered the standard drug for the treatment of colon cancer [31]. The pyrimidine anti-metabolite of 5-FU is a chemotherapeutic agent used for various types of solid cancers, alone or in combination with an adjuvant such as leucovorin (LV) [32]. The 5-FU group induced a significant decrease in leucocyte counts in the tumor xenograft mice. Whereas, the MFE treatment complemented the immune suppression induced by 5-FU.

MFE significantly increased the proliferation of ConA and LPS stimulated splenocyte in the tumor xenograft mice. The results indicate that MFE might initiate a potential activation of T and B lymphocytes and enhance the humoral and cell-mediated immunity in tumor xenograft mice.

When tumor cells invade healthy tissue, the immune system can counterattack [18]. The immune system plays an important role in the fight against cancer. Many reported studies have shown that tumoricidal effects of natural products are mediated by the activation of the immune response. The basis of cancer immunotherapy is to boost the body’s natural immune defenses against the tumors. Recently the antitumor activity of natural products through the immune response has been focused on developing chemotherapeutic agents [33]. The host contains a population of naturally occurring lymphocyte-like cells that are cytotoxic to tumor cells. NK lymphocytes are considered important players at inhibiting tumors [34]. NK cells do not need to recognize antigens on the immune cells. Whereas, CTL was promoted because it recognizes antigens in the surface of immune cells [35,36]. MFE significantly increased the NK cells and CTL activities in the tumor xenograft mice, suggesting that MFE could enhance specific and non-specific cytotoxicity against tumor cells.

Macrophages, NK cells and even NK T cells themselves can produce IFN-γ during the innate immune response and exhibit immune stimulatory properties and anti-tumor activities that can inhibit cancer cell proliferation and tumor angiogenesis [37,38]. The increase in the levels of IFN-γ secretion from splenocytes in MFE treated mice could explain the antitumor properties of MFE.

Th1 T cells provide both IFN-γ for macrophage activation and B cell help to produce IgG subclasses for the opsonization of pathogens. MFE promoted an immune response involving the Th1 type response in the tumor xenograft mice, a response that is related to the significant increase of IgG2a levels. Further studies are necessary to confirm whether MFE could stimulate the humoral immune response caused by the model system using TLR4 knockout mice [39].

## 4. Experimental Section

### 4.1. Chemical and Reagents

Dulbecco’s Modified Eagle’s medium (DMEM), Roswell Park Memorial Institute medium 1640 (RPMI) and fetal bovine serum (FBS) were obtained from GIBCO (Grand Island, NY, USA). A nitric oxide (NO) detection kit was obtained from INTRON Biotechnology (Sungnam, Korea). Trizol was obtained from Invitrogen (Carlsbad, CA, USA). Interleukin (IL)-12, interferon-γ (IFN-γ) and tumor necrosis factor-α (TNF-α) were obtained from R&D Systems (Minneapolis, MN, USA). A PCR relative kit, small interfering siRNA and primer were obtained from Applied Biosystems (Franklin Lakes, NJ, USA). Penicillin, streptomycin, neutral red, 3-(4,5-dimethylthiazol-2-yl)-2,5-diphenyltetrazolium bromide (MTT), lipopolysaccharide (LPS), cholera toxin (CT), and all other chemicals were obtained from Sigma-Aldrich (St. Louis, MO, USA).

### 4.2. Cell Culture and Animal Care

RAW264.7 (TIB-71) and CT26 (CRL-2638) were obtained from the American Type Culture Collection (Manassas, VA, USA). Cells were grown in DMEM or RPMI medium supplemented with 10% heat-inactivated FBS, 100 U/mL of penicillin, and 100 μg/mL of streptomycin. Cells were grown at 37 °C in a humidified 5% CO_2_ incubator. Mice were housed in specific pathogen-free (SPF) conditions at 21 to 24 °C and between 40% and 60% relative humidity with a 12 h light-dark cycle. All animals were acclimatized for at least 1 week prior to the start of experiments. All studies were performed in accordance with the guide for animal experimentation by Wonkwang University and approved by the university’s institutional animal care and use committee (Approval No. WKU11-43).

### 4.3. Extract of Morus alba Fruits

Dried *M. alba* fruits were purchased from a local herbal market in Jeonbuk, Korea. The fruits were pulverized into powder and extracted twice with hot water (70 °C) for 3 h. The solvent was removed under reduced pressure in a RV10 rotary evaporator (IKA, Staufen, Germany) to yield *M. alba* fruit hot water extract (MFE; 59.3%, *w*/*w*). The extract was dried to a powder and kept in a closed container until use. To avoid variations in activity for different preparations, enough extract was obtained in one batch for use throughout the experiment. The content of the marker pyrrole alkaloid, 2-formyl-5-(methoxymethyl)-1-*H*-pyrrole-1-butanoic acid in MFE was quantitated using high performance liquid chromatography (HPLC).

### 4.4. HPLC Estimation of Pyrrole Alkaloid

**Figure 5:**
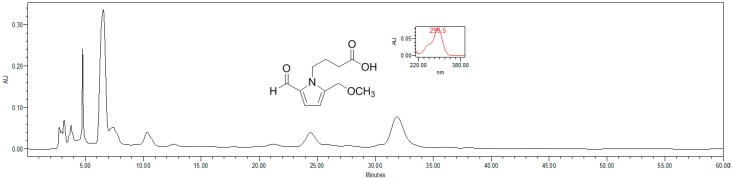
HPLC analysis of *Morus alba* L. fruits and the chemical structure of pyrrole alkaloid.

For HPLC analysis, MFE was dissolved in HPLC grade methanol to obtain a 5 mg/mL concentration, and filtered through a 0.45 μm syringe filter. These solutions were further used for HPLC analysis. Reference standard pyrrole alkaloid was prepared as 0.1 mg/mL of 2-formyl-5-(methoxymethyl)-1-*H*-pyrrole-1-butanoic acid in 1 mL of HPLC grade methanol. HPLC analysis was performed as described previously. The HPLC system consisted of a model 515 pump and model 717 autosampler (Waters, Beverly, MA, USA). Reverse phase separation was performed at room temperature using Gemini-NX (5 μm, 10.0 mm × 150.0 mm; Phenomenex, Torrance, CA, USA). The mobile phase consisted of 60% water containing 0.5% H_3_PO_4_ and 40% acetonitrile. The flow rate was kept at 1 mL/min and the peak was detected at 300 nm. The marker compound was identified by comparing the UV spectrum and retention time. The estimation of pyrrole alkaloid content in aqueous extracts with the marker compound pyrrole alkaloids 2-formyl-5-(methoxymethyl)-1-H-pyrrole-1-butanoic acid was performed for standardization (Figure 5).

### 4.5. Proliferation Assay

Cell proliferation was evaluated by the MTT assay. Cells (2 × 10^5^/well) were incubated with LPS, Concanavalin A (ConA), or MFE (10–100 μg/mL) in wells of a 96-well plate. MTT was added to each well to a final concentration of 1 mg/mL. During incubation for 3 h, formazan crystals were formed by the action of mitochondrial enzymes in viable cells. The crystals were solubilized in dimethyl sulfoxide. The absorbance was measured at 590 nm using a microplate reader (Molecular Devices, Sunnyvale, CA, USA).

### 4.6. NO and Cytokine Assays

Cells (2 × 10^5^/well) were incubated with LPS or MFE (10–100 μg/mL) in 96-wells plate. After 24 h the plate was centrifuged at 3000 rpm for 5 min, and the supernatants were collected for the detection of TNF-α and IFN-γ levels using commercial ELISA kits. Nitrite accumulation in the culture medium as an indicator of NO production was determined using a Griess reaction kit.

### 4.7. Antibody Inhibition Experiments

Peritoneal macrophages were plated into wells of 96-well culture plates and pre-treated with monoclonal antibody (mAb) to TLR2, TLR4, TLR6, SB203580, or pyrrolidinedithiocarbamate (PDTC) for 2 h. MFE was added and incubated for 24 h. TNF-α in culture supernatants was analyzed by an ELISA kit according to the manufacturers’ directions (R&D Systems, Minneapolis, MN, USA).

### 4.8. siRNA Preparation and Transfection

siRNA duplexes with the following gene-specific sense sequences were used: TLR2 (Mm. 87596), TLR4 (Mm. 38049), MyD88 (Mm. 213003), and toll/IL-1 receptor domain-containing adaptor inducing IFN-β (TRIF; Mm. 149280). Each sequence along with 100 nM of the specific siRNA was transfected into RAW 264.7 cells using Lipofectamin 2000 (Invitrogen, Carlsbad, CA, USA) according to the manufacturer’s protocol. Cells were incubated at 37 °C in a CO_2_ incubator for 24 h for gene knockdown.

### 4.9. Western Blots

Total cellular protein (50 μg) was resolved by polyacrylamide gels containing 10% sodium dodecyl sulfate-polyacrylamide gel electrophoresis (SDS-PAGE) and transferred to nitrocellulose membranes. After blocking, the membranes were incubated with the target antibody. Horseradish peroxidase secondary antibody conjugated to IgG was used. The blots were probed using the ECL Western blot detection system (GE healthcare, Maple Grove, MN, USA) as instructed by the manufacturer.

### 4.10. Macrophage-Mediated Cytotoxicity

Macrophages (effector cells; 1 × 10^5^ cells/well) were plated into a 96-wells plate and incubated with various MFE concentrations for 18 h at 37 °C in a 5% CO_2_ incubator. The effector cells were washed with RPMI-FBS to remove the MFE and co-incubated with the CT26 (target cells; 1 × 10^4^ cells/wells). MTT solution (1 mg/mL) was added to each well and the plate was incubated for another 4 h prior to the MTT assay. The cytotoxicity was expressed follows (Equation (1)) [40]:

(1) $$1-\frac{\text{O.D. of }{\left(\text{Target cell + effector cell}\right)}_{\text{sample}}-\text{O.D. of }{\left(\text{only effector cell}\right)}_{\text{sample}}}{\text{O.D. of }{\left(\text{only Target cell}\right)}_{\text{blank}}}\times 100$$

### 4.11. Treatment and Drug Administration

Female balb/c mice (*n* = 40) were shaved on their right flank and given a subcutaneous injection of 2 × 10^5^ CT26 cells to induce tumor formation. Control mice did not receive treatment. Cancer cells were allowed to grow into visible masses for 10 days, after which animals were divided into four groups of 10 mice each: the normal group (no treatment and no cancer), the control group (cancer), the 5-FU-treated group (50 mg/kg) and the 5-FU + MFE treated group (100, 300 mg/kg). The daily MFE administration of the mice in our study, 100 and 300 mg/kg body weight, was equivalent to an administration of approximately 8.1 and 24.3 mg/kg human body weight respectively, when calculated on the basis of normalization to body surface area under the recommendations of the USA Food and Drug Administration and Reagan-Shaw *et al.* [41]. 5-FU treatment was administered by one intraperitoneal injection and MFE was orally administered daily for 3 weeks. Animals were sacrificed after the end of the experiment. The tumor and visceral organ weights were excised and measured.

### 4.12. Evaluation of Tumor Volume, Leukocyte Count, Tumor Weight, and Organ Weight

Body weight of each mouse was determined every day by a single observer. Tumor growth was monitored every 2 days by measuring two perpendicular tumor diameters with a caliper. Tumor volume (*v*) was calculated as: *v* = [(A − B)/A] × 100%, with A and B being the smallest and largest superficial diameter, respectively. Animals were sacrificed 21 days after inoculation. After the mice were sacrificed the tumor, organ weights, and leucocyte level were measured.

### 4.13. Assay of NK Cells and CTL Activity

NK cells and CTL activities were measured as described in the previous study [42]. Splenocytes were collected from the tumor xenograft mice as the effector cells, and were added at 1 × 10^6^ cells/well. K562 cells (NK target) and CT26 cells (CTL target) were seeded in a 96-well, flat bottom microtiter plate at 2 × 10^4^ cells/well to give an E/T ratio 50:1 in RPMI 1640 complete medium. The plate was incubated for 24 h at 37 °C in a 5% CO_2_ atmosphere. MTT solution was added to each well and the plate was incubated for another 4 h and subjected to the MTT assay. The percentage of target cells killed (% lysis) was calculated as Equation (2):

(2) $$\frac{{\text{O.D.}}_{\text{T}}-({\text{O.D.}}_{\text{S}}-{\text{O.D.}}_{\text{E}})}{{\text{O.D.}}_{\text{T}}}\times 100$$

where O.D._T_ is the optical density value of target cells control, O.D._S_ is the optical density value of test samples, and O.D._E_ is the optical density value of effector cells control.

### 4.14. Measurement of Antigen-Specific Antibody

Antigen-specific IgG, IgG1, and IgG2a antibody in serum were detected by an indirect ELISA as described in the previous study [43]. The absorbance value was measured with an ELISA reader at 490 nm. Data were expressed as the mean absorbance of the samples minus the mean absorbance of the normal control.

### 4.15. Statistical Analysis

The data were expressed as mean ± SD and were examined for their statistical significance of difference with analysis of variance and Student’s *t*-test. A *p*-value <0.05 was considered to be statistically significant.

## 5. Conclusions

**Figure 6:**
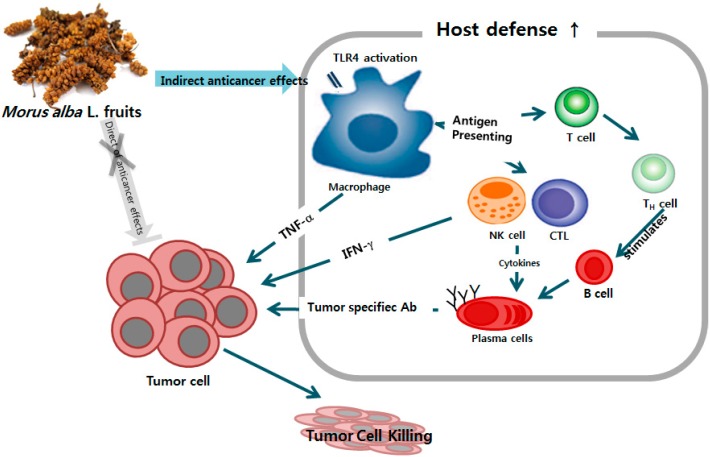
Summary of the effects of *Morus alba* L*.* fruit extract against cancer.

MFE stimulated the production of cytokines via TLR4 and the tumoricidal activity of macrophages. Macrophage activation was induced through TLR4-mediated NF-κB and MAPK signaling pathways, which initiated the release of cytokines like TNF-α and IL-12 and effector molecules like NO. MFE not only significantly inhibited the growth of colon cancer cells, but markedly increased spleen weight, splenocyte proliferation, activity of NK cells and CTL activity, the level of IFN-γ, and serum antigen-specific antibody levels in xenograft mice. All these results indicate the possibility that MFE may be able to improve both specific and nonspecific cellular and humoral immune response (Figure 6). Thus, MFE is implicated in the defense against cancer by enhancing immune system function through TLR4 signaling-mediated activation of cytokines and immune cells. *Morus alba* L. fruits should be further explored as a potential anti-tumor immunomodulatory agent.
